# Medroxyprogesterone acetate and meningioma: a global issue

**DOI:** 10.3389/fgwh.2025.1470539

**Published:** 2025-03-26

**Authors:** Noémie Roland, Sébastien Froelich, Alain Weill

**Affiliations:** ^1^ EPI-PHARE Scientific Interest Group Department (French National Agency for the Safety of Medicines and Health Products, and French National Health Insurance), Saint-Denis, France; ^2^ Department of Neurosurgery, Lariboisière University Hospital, Paris-Cité University, Assistance Publique-Hôpitaux de Paris, Paris, France

**Keywords:** depot medroxyprogesterone acetate, contraception, meningioma, progestogen, public health

## 1 Introduction

Depot medroxyprogesterone acetate (DMPA) is a widely-prescribed injectable progestogen contraceptive used by 74 million women each year around the world (1). DMPA is particularly popular for its high effectiveness and ease of administration (2). Indeed, this contraception consists of a medroxyprogesterone acetate 150 mg/ml intramuscular injection or a 104 mg/0.65 ml subcutaneous injection every 3 months (3). DMPA's usage varies greatly from one country to another: from 1.8% of women aged 15–49 in high-income countries to 8.7% in low-income countries (1). Inexpensive, DMPA is particularly widespread in countries with a strong family planning policy, like many countries in South-Eastern Asia (13% of women of Southeast Asia use DMPA, i.e., about 22 million women/year in 2019) (1, 4). In Indonesia, it is the contraception used by one quarter of women (17 million women, which makes this country the biggest user of this contraception), and by one woman out of five in Myanmar (2.5 million), one woman out of 10 to 20 in Thailand (1.5 to 2 million), Cambodia (0,3 million), Laos (0,2 million), and one woman out of 30 in the Philippines (0,9 million) and Malaysia (0,3 million) (1, 5, 6). In Africa, Malawi (33.6% of women, 1.1 million), Ethiopia (21% of women, 4.6 million) and South Africa (23.4% of women, 3.6 million) are the most concerned. Lastly, 1% of Australian women were concerned by this contraception in 2015, and in the USA, 25.4% of women aged 15–44 years have ever used DMPA in their life (2.6% of American women/year, 1.7 million) (7).

However, DMPA has significant and sometimes serious adverse side effects, such as weight gain, menstrual irregularities, changes in mood, osteoporosis, and arteriovenous thrombosis disorders. In addition to these known side effects, the risk of meningioma has been added by international scientific publications for DMPA, as added previously for other progesterone-derived drugs.

## 2 Risk of meningioma

### 2.1 Progressive evidence of a causal relationship between progestogens and meningioma

As early as 2007, two case reports (in Italy and France) reported the first cases of meningioma in women exposed to a progestogen with strong antiandrogen effects, cyproterone acetate, suggesting that this progestogen is a causal factor of a particular histopathological tumor entity (8, 9). The French team also noted stabilization of the tumor after the cessation of cyproterone acetate treatment (9).

In 2019, a nationwide pharmaco-epidemiological study conducted by the French National Health Insurance confirmed the strong dose-response relationship between the use of cyproterone acetate and meningiomas requiring surgery or radiotherapy (10). Indeed, the risk was multiplied by 20 for a cumulative dose over 60 g (i.e., 5 years of treatment). In the aftermath of this study, the European Medicines Agency (EMA) reassessed the benefit/risk ratio of cyproterone acetate and recommended restricting its prescription and regular MRI screening. Cyproterone acetate is marketed in high doses in Australia, Indonesia, Thailand and New Zealand but not currently marketed in the United States. This progestogen treatment is also widely used in many countries as antiandrogen cross-sex hormone treatment for transgender women with a well-identified risk of intracranial meningioma (11).

Then, in 2021, studies found a strong association between the risk of meningioma and the use of two other potent progestogens, nomegestrol and chlormadinone acetate, mainly prescribed for the hormonal treatment of menopause in Europe. In Asia and Oceania, nomegestrol acetate (2.5 mg in combination with estradiol 1.5 mg) is only marketed in Australia, Malaysia and New Zealand, whereas chlormadinone acetate is not marketed. These drugs have never been approved in the US. The magnitude of meningioma risk with nomegestrol and chlormadinone was less than that of cyproterone acetate, although still high (12–15). In July 2022, the EMA recommended that high-dose formulations of nomegestrol and chlormadinone acetate be used at the lowest possible dose over the shortest possible period.

For these three progestogens—cyproterone, chlormadinone and nomegestrol acetate -, European drug regulators have recognized the causal nature of the association between exposure and intracranial meningiomas.

### 2.2 About 6-fold increase in the risk of meningioma for DPMA

In a recent study in France (12), where DMPA is not frequently used, an excess risk of intracranial meningioma linked to its long-term use (≥1 year) has been found at the dose of 150 mg [OR = 5.6 (2.2–14.4)] (12), leading to fears of a significant prevalence of intracranial meningiomas where women are highly exposed to DMPA. If this result had been isolated internationally, it could have constituted a weak signal that would have to be further analyzed and, if necessary, confirmed by other studies. However, there is already a great deal of converging evidence in favor of a very strong association between DMPA contraception and meningiomas.

First, meningiomas that occur during DMPA exposure seem to share common characteristics with meningiomas associated with other progestogens [cyproterone, nomegestrol and chlormadinone acetates (10, 14)]: high progesterone- and low estrogen-receptors expressions, frequent existence of several meningiomas at the time of diagnosis (meningiomatosis), preferential location in the anterior and/or middle part of the base of the skull (10, 12–15), regression of volume on cessation of progestogen treatment (16–18), predominance of transitional sub-types, and a specific mutational landscape with a high rate of PIK3CA mutation and low rate of NF2 mutation (19). The characteristic location in the skull base makes surgery more difficult, with more frequent incomplete resections, leading to more sequelae, recurrences and re-operations.

Secondly, a first case-report in 2000 (20), a review of case reports in the USA published in 2023 (21), a large case-control in the USA published in September 2024 (22), and several studies carried out and Indonesia (seven studies, including only three medline-indexed), in which one quarter of women taking contraception use DMPA, have assessed a risk between this contraceptive exposure and intracranial meningiomas (6, 23–29). These studies have been somewhat overlooked, despite providing us with valuable epidemiological data Table 1.

**Table 1:** Available observational studies on medroxyprogesterone acetate and risk of meningioma.

Authors, year of publication	Accessibility [journal]	Main elements	Strengths	Limitations
Hensiek et al. (20)	Medline-indexed [British Journal of Neurosurgery]	Case-report (UK) 2000 44-year-old man with a surgery for an olfactory groove meningioma after 4 years of medroxyprogesterone therapy (100 mg/tds) for renal cell carcinoma	First report	Limited to one case
Supartoto et al. (24)	Medline-indexed [International Journal of Ophthalmic Pathology]	Case-control study (Indonesia) 2010–2014 40 orbitocranial meningioma cases (21 exposed to DMPA) OR 2.47(CI: 95%; 1.08–5.64)^a^ for the use of hormonal contraception containing progesterone	High exposure to DMPA in Indonesia Integration of the duration of exposure Adjustment for education level, age of menarche, length of menstrual cycle and number of parity	Regional (3 hospitals, Yogyakarta area) Limited to orbitocranial meningioma Low number of cases Study of exposure to global hormonal contraception (not specific to DMPA, but high exposure to DMPA in Indonesia)
Dewata et al. (23)	Not medline-indexed [Advanced Science Letters]	Case-control study in Soetomo hospital (Indonesia) 2012–2016 212 cases of operated meningiomas, 155 exposed to DMPA OR 3.13 (CI: 95%; 2.03–4.85)	High exposure to DMPA in Indonesia Integration of the duration of exposure	Monocentric Low number of cases
Wahyuhadi et al. (25)	Not medline-indexed [Majalah Obstetri & Ginekologi]	Case-control study in Soetomo hospital (Indonesia) 2012–2013 101 cases of operated meningiomas, 84 exposed to DMPA (vs. 14 controls)	High exposure to DMPA in Indonesia Integration of the duration of exposure Rate of meningiomas exposed to DPMA > 80% [recalculated OR > 30, 18-fold increased odds of meningioma for contraceptive use over 10 years (relative to no use)]	Monocentric Low number of cases
Anggraeni et al. (26)	Not medline-indexed [Biomedical Journal of Indonesia]	Cross-sectional study (Indonesia) One year (2018) 67 cases of operated meningiomas	High exposure to DMPA in Indonesia	Monocentric Low number of cases Study of exposure to global hormonal contraception (not specific to DMPA)
Dustur et al. (27)	Not medline-indexed [Pharmacognosy Journal]	Case-control study in Soetomo hospital (Indonesia) 2015–2019 452 cases of operated meningiomas, 101 exposed to DMPA	High exposure to DMPA in Indonesia Integration of the duration of exposure (< or >10 years) Age and histological analyses	Monocentric
Malueka et al. (29)	Medline-indexed [Asian Pacific Journal of Cancer Prevention]	Indonesia: 99 cases of meningiomas (77 exposed to hormonal contraception) Retrospective data from medical records of all histologically confirmed meningioma patients in Dr. Sardjito General Hospital, Yogyakarta, Indonesia 2019-early 2021 OR hormonal use (as a whole): 2.57 (1.04–6.37)	High exposure to DMPA in Indonesia Location, age, symptoms, histological analyses	Monocentric Low number of cases Study of exposure to hormonal contraception (not specific to DMPA)
Rusdi et al. (28)	Medline-indexed [Natural volatiles & essential oils Journal]	Cross-sectional study at dr. Soetomo hospital, Surabaya (Indonesia) 2015–2020 206 cases of operated meningiomas, 67 exposed to DMPA	High exposure to DMPA in Indonesia Histological analyses	Monocentric Low number of cases
Abbou-Al-Shaar et al. (21)	Published abstract [Journal of Neurological Surgery Part B: Skull Base]	Retrospective review of meningioma patients with a history of DMPA use treated in USA at the University of Pittsburgh Medical 2014–2021 25 cases of meningiomas Tumor shrinkage occurred among half of the patients who were instructed to cease their use of DMPA	Location, age, symptoms, histological analyses many specific skull base locations and multiple meningiomas	Monocentric Low number of cases
Roland et al. (12)	Medline-indexed [BMJ]	National matched (5:1) case-control study SNDS database 2009–2018 18,061 cases of operated meningiomas, 9 exposed to DMPA (0.05%) vs. 11/90,305 controls (0.01%) OR: 5.55 (2.27–13.56)	National Exhaustive real-world data 12 years proper consideration of confounding factors (exclusion criteria), negative and positive controls Analyses by durations	Low exposure to DMPA No clinical nor histological details
Griffin (22)	Medline-indexed [Cancers]	Large matched (10:1) nested case-control study IBM MarketScan database 2006–2022 87,455 cases of cerebral meningiomas, 813 exposed to DMPA injectable aOR any duration DPMA: 1.68 (1.50–1.87); aOR exp >3 years 3.24 (2.49–4.21)	High number of cases and duration of exposure Oral MPA analysis of (no risk) Spinal meningioma analysis of (no risk)	Limited to private insurance No clinical nor histological details

^a^ OR, odds ratio. OR are mentioned if they are presented in the study.

We searched PubMed for original research in English from Jan 1, 2000, to October 1, 2024, with the terms “medroxyprogesterone” and “meningioma” and we found 12 articles. We excluded 9/12 articles that considered medroxyprogesterone as a treatment for meningioma We then searched google scholar from Jan 1, 2000, to October 1, 2024, with the terms “medroxyprogesterone”, “contraception” and “meningioma” and without the words “cyproterone” and “megestrol”, and we found 166 articles. We excluded articles that considered medroxyprogesterone acetate as a treatment for meningioma or not relevant to the specific subject, and kept 11 articles (8 more than Pubmed research).

In the United-States, Abou-Al-Shar et al. first performed a retrospective review of meningioma patients with a history of DMPA use treated at the University of Pittsburgh Medical center between 2014 and 2021 (21). They included 25 women with a mean age of 46 years suffering from intracranial meningiomas [a total of 49 meningiomas (1 to 6 per woman)] and exposed to DMPA chronic use. The mean duration of use was 15 years [6 to 26 years] and more than two thirds of patients had at least one skull base meningioma located in the spheno-cavernous, spheno-orbital, or orbital region. Interestingly, five non-operated patients showed clear signs of tumour shrinkage after stopping DMPA injections. Moreover, recently, using US private insurance data, a large case-control study showed that DMPA exposure was associated with meningioma [480 cases of cerebral meningioma exposed to DMPA, OR: 1.68 (95% CI: 1.50–1.87)] (22). This association was dose-dependent (OR DPMA > 3 years: 3.24 (2.49–4.21). This association was not found with oral medroxyprogesterone [OR 0.99 (0.94–1.04)], nor with spinal meningiomas (22).

Then, some monocentric Indonesian studies have assessed hormonal contraceptive exposure in surgical records of meningiomas (from 47% for Anggraeni et al. in 2018 (26) to 96% for Dustur et al. in 2015–2019 (27). Some studies detailed the prior use of hormonal contraceptives et found specific history of DMPA exposure (from 36% of meningioma patients for Rusdi et al. (28) to 83% for Dustur et al. (27). Lastly, two case-controls studies assessed the dose-response association between meningioma risk and DMPA use. Wahyuhadi et al. found that DMPA has a higher risk rate of meningioma compared with other hormonal contraceptives (25): 84/101 (83.2%) cases were exposed among the cases group (i.e., operated on meningiomas), and 14/101 (13.9%) were exposed among the controls (non-meningioma patients who had undergone contrast head CT-scan). Even though cases and controls in this study were not completely similar in terms of age, these results are particularly concerning. Lastly, Dewata et al. found a specific risk of meningioma among women exposed to DMPA (155 DMPA users/212 cases) of 3.13 (CI: 95%; 2.03–4.85) with a dose-cumulative effect: the longer the duration of exposure to DMPA, the higher the risk for meningioma [duration of 10 to 15 years: OR 2.33 (CI: 95%; 1.31–4.15), and more than 15 years: OR 4.45 (CI: 95%; 2.35–8.35)] (23).

## 3 Discussion

### 3.1 A plea for a global awareness

Using the incidence of meningioma among women aged 35–44 year-old from Wiemels et al. (30), we can assess that a risk of meningioma multiplied by 1.68 [OR from Griffin, 2024 (22)] to 5.55 [Roland et al. (12)] would lead to a number of symptomatic and/or surgical meningiomas attributable to DMPA exposure of more than 2,800 to more than 18 000 worldwide each year.

The magnitude of the risk of meningioma between that associated with the use of cyproterone acetate and that associated with DMPA is, of course, not the same. Nevertheless, DMPA is much more widely prescribed than CPA, and the contraceptive can be used without interruption for periods of over 10 years. With the arrival of the subcutaneous form, we can expect to see even more prescriptions for DMPA worldwide than before, as it could be self-administrated, although with a smaller dose (31). As the subcutaneous form is not available in France, we have not been able to carry out meningioma risk studies with this specific dosage. Countries with access to these data should take up this subject to assess the associated risk of meningioma with 104 mg/0.65 ml form.

DMPA may influence the cellular proliferation of meningioma cell lines; however, the molecular mechanisms underlying this process fall beyond the scope of this viewpoint article. While a potential causal relationship between DMPA use and meningioma formation cannot be ruled out, definitive experimental evidence remains lacking. Notably, some studies on animal models of meningiomas have reported an overexpression of progesterone receptors in subjects that developed these tumors (32, 33). Given the potential clinical implications, specialized molecular research teams should further investigate this topic to clarify the underlying mechanisms.

While DMPA is presented as a lever for equitable access to contraception (34), particularly since the COVID crisis or in the post-Roe era (35), women and prescribers must be aware of the risk of meningioma in order to make an informed choice of contraception. In our experience, neurological monitoring should be the rule in the event of DMPA prolonged treatment (more than 10 years) and in patients over 40 years of age to detect a meningioma as early as possible. In case of meningioma discovery, DMPA must be discontinued, as this may lead to tumor regression and avoid the need for surgery.

Since these DPMA-exposed meningiomas occur in countries with a low socio-economic level where access to brain imaging for neurological symptoms could be very complicated and neurosurgery less accessible, it is likely that many diagnoses are ignored or that skull-base surgery is rejected because of its complexity, the vital risk and the neurological prognosis. Similarly, in high-income countries, DMPA particularly affects vulnerable populations or those suffering from known inequalities in access to healthcare (e.g., DMPA users are disproportionately non-White in the USA (34), and DMPA is also known to prescribed to migrants and people with mental conditions (36). Thus, even in countries with extensive healthcare networks, the utmost vigilance is also called for.

### 3.2 Conclusion

The meningioma risk arising from the prolonged and ongoing use of DMPA should be the subject of epidemiological studies in all the countries concerned, especially cohort or case-control studies using claims data, such as the one carried out recently in the USA (22). Priority should be given to targeting people with the least access to health care facilities: disadvantaged populations in developed countries, for whom DMPA is often prescribed (using public rather than private insurance databases, for example), or to larger-scale, better-conducted case-control studies in neurosurgical centers from disadvantaged countries than those carried out in Indonesia to date. Lastly, assessing the risk associated with the specific dose prescribed subcutaneously, and the risk associated after treatment discontinuation are also important issues. The benefit-risk balance and alternatives also need to be discussed.

We call on the international scientific community to mobilize in the face of this legitimate public health concern on the sensitive but essential subject of contraception. Assessing the risk of meningioma among DMPA users, especially in countries with low socio-economic levels, has become a global public health necessity.
